# Notes on Treatment

**Published:** 1908-05-09

**Authors:** 


### NOTES ON TREATMENT.

**Pain in Osteo-Arthritis.**

A few months ago inquiry was made in one of the medical papers for a method of treating pain in osteo-arthritis. Amongst the answers given no mention was made of ichthyol. Ichthyol is not an elegant remedy, but it may give great relief when applied externally. One part of ichthyol to two parts of olive oil is generally of sufficient strength.

**Chronic Urticaria in Children.**

Cases are occasionally met with where urticaria in young children has existed since early infancy. Dyspepsia may be treated, tonics given, external palliatives applied, but the troublesome complaint continues. In such cases small doses of calomel, combined with salicin or salicylate of bismuth, may do good, or great benefit may be derived from ichthyol given internally in a mixture containing glycerine with some preparation of ginger to cover the taste.

